# Neurovascular complications of antiphospholipid syndrome: a narrative review

**DOI:** 10.1055/s-0044-1793932

**Published:** 2024-12-10

**Authors:** George Nilton Nunes Mendes, Alessandra Braga Cruz Guedes de Morais, Laura Catherine Gioia, Grégory Jacquin, Alexandre Y. Poppe, Felipe Hideki Soga, João Brainer Clares de Andrade

**Affiliations:** 1 Université de Montréal, Centre Hospitalier, Programme de Santé Neurovasculaire, Montréal Québec, Canada.; 2 Université de Montréal, Centre Hospitalier, Centre de Recherche, Axe Neurosciences, Montréal Québec, Canada.; 3 Universidade Federal de São Paulo, São Paulo SP, Brazil.; 4 Université de Montréal, Faculté de Médecine, Département de Neurosciences, Montréal Québec, Canada.; 5 Centro Universitário São Camilo, São Paulo SP, Brazil.; 6 Hospital Israelita Albert Einstein, São Paulo SP, Brazil.

**Keywords:** Antiphospholipid Syndrome, Stroke, Cognition, Venous Thrombosis, Complications, Síndrome Antifosfolipídica, Acidente Vascular Cerebral, Cognição, Trombose Venosa, Complicações

## Abstract

**Background** Antiphospholipid syndrome (APS) is a systemic autoimmune disorder characterized by thrombosis, pregnancy complications, and other nonthrombotic manifestations in the presence of antiphospholipid antibodies. Neurovascular complications, including ischemic stroke, cerebral venous thrombosis and cognitive impairment, pose significant challenges in management.

**Objective** To comprehensively review relevant and updated clinical aspects of neurovascular manifestations of APS.

**Methods** We conducted a narrative review using the PubMed, EMBASE, and Cochrane Library databases with medical terms related to APS and its neurovascular manifestations. English-language studies, published between January 1, 2015, and March 2024, were included. Key publications outside this timeframe were also considered. Studies with higher levels of evidence, such as randomized controlled trials and meta-analyses, were prioritized for inclusion.

**Results** Stroke is a prevalent complication in APS, with arterial thrombosis being a predominant mechanism. Despite recent trials, direct oral anticoagulants (DOACs) have not shown superiority over vitamin K antagonists (VKAs) for secondary prevention in this population. Cerebral venous thrombosis (CVT), although rare, can also occur in APS, and while DOACs have shown promise as a treatment in a general population, caution is warranted due to potential harm. Cognitive impairment affects a considerable proportion of APS patients, with thrombotic and nonthrombotic mechanisms contributing to its pathophysiology. Future research should focus on optimal management strategies for cognitive impairment and the efficacy of anticoagulation and immunosuppression.

**Conclusion** Understanding the complex interplay of neurovascular manifestations in APS is essential for guiding clinical decisions and improving patient outcomes. Despite advancements, some challenges remain in establishing effective preventive and treatment measures, highlighting the need for further research in this field.

## Resumo

**Antecedentes** A síndrome antifosfolipídeo (SAF) é uma doença autoimune sistêmica caracterizada por trombose, complicações na gravidez e outras manifestações não trombóticas na presença de anticorpos antifosfolipídicos. Complicações neurovasculares, incluindo acidente vascular cerebral isquêmico, trombose venosa cerebral (TVC) e comprometimento cognitivo, representam desafios significativos no manejo.

**Objetivo** Revisar de forma abrangente e atualizada os aspectos clínicos relevantes das manifestações neurovasculares da SAF.

**Métodos** Foi realizada uma busca nas bases de dados PubMed, EMBASE e Cochrane, utilizando termos médicos relacionados à SAF e suas manifestações neurovasculares. Estudos em inglês publicados entre 1° de janeiro de 2015 e março de 2024 foram incluídos. Publicações relevantes fora deste período também foram consideradas. Estudos com níveis mais elevados de evidência, como ensaios clínicos randomizados e metanálises, foram priorizados para inclusão.

**Resultados** O acidente vascular cerebral é uma complicação prevalente na SAF, com a trombose arterial sendo um mecanismo predominante. Apesar de ensaios recentes, os anticoagulantes orais diretos (ACODs) não demonstraram superioridade sobre os antagonistas da vitamina K (AVKs) para a prevenção secundária nesse grupo. A trombose venosa cerebral (TVC), embora rara, também pode ocorrer na SAF, e embora os ACODs tenham mostrado promessa em seu tratamento na população geral, deve-se ter cautela devido ao possível dano. O comprometimento cognitivo afeta uma proporção considerável dos pacientes com SAF, com mecanismos trombóticos e não trombóticos contribuindo para sua fisiopatologia. Pesquisas futuras devem se concentrar em melhores estratégias de manejo para o comprometimento cognitivo e na eficácia da anticoagulação e imunossupressão.

**Conclusão** Compreender a complexa interação das manifestações neurovasculares na SAF é essencial para orientar decisões clínicas e melhorar os resultados dos pacientes. Apesar dos avanços, alguns desafios permanecem no estabelecimento de medidas preventivas e terapêuticas eficazes, destacando a necessidade de mais pesquisas nesse campo.

## INTRODUCTION

Antiphospholipid syndrome (APS) is a known systemic disease classically described as an autoimmune development of arterial, venous, or microvascular thrombosis, pregnancy complications, or nonthrombotic manifestations with persistent antiphospholipid antibodies. Recent diagnostic criteria were published in 2023, including clinical and laboratory findings with different weights for each specific component. 1

The main antiphospholipid antibodies (APA) are lupus anticoagulant, IgG/IgM anti-cardiolipin, and IgG/IgM anti–β2 glycoprotein I antibodies. 2 While anti-phosphatidylserine-prothrombin, phosphatidic acid, phosphatidyl-ethanolamine, phosphatidyl-glycerol, phosphatidyl-inositol, phosphatidylserine, and annexin V are related to APS and seem to play a role in clinical practice, they are not considered as part of its diagnostic criteria. 3 This syndrome may be primary, when isolated, or secondary, when associated with a concomitant systemic disease (most commonly systemic erythematous lupus, or SLE). 3

The pathophysiology of APS involves several interconnected mechanisms, of which the most central are APA, which target phospholipids and associated proteins, disrupting coagulation and promoting thrombosis. These antibodies also induce platelet activation, enhancing clot formation and stability, and interact with endothelial cells, increasing the expression of adhesion molecules and tissue factors, leading to a proinflammatory and prothrombotic state. Furthermore, APS are associated with increased oxidative stress markers and reduced antioxidant capacity, contributing to vascular damage and thrombosis. Additionally, anti-phospholipids (aPLs) activate the complement system, which plays a crucial role in the inflammatory response and thrombus formation among APS patients. 4 5

**Figure 1:**
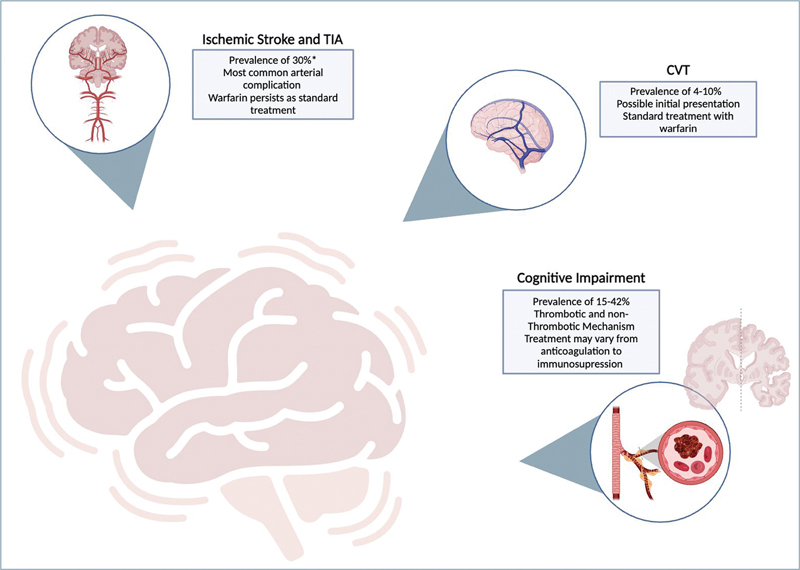
Neurovascular complications of APS. **Abbreviations:** APS, antiphospholipid syndrome; TIA, transient ischemic attack.

Neurovascular complications have been described in APS patients as an important cause of morbidity and mortality. 6 Given the clinical complexity and limited treatment options for such complications, this narrative review seeks to explore clinical decision-making in that ( Figure 1 ), with an emphasis on possible treatments and clinical outcomes.

## METHODS

### Search approach

For this literature review, we conducted an exhaustive search of the PubMed, EMBASE and Cochrane databases, during the publication period of January 1 ^st^ , 2015, to February 29, 2024, including a spectrum of original research and review articles employing a blend of pertinent keywords and medical terminologies (MeSH) to capture pertinent studies. Search terms included *antiphospholipid syndrome and stroke* , *antiphospholipid syndrome and neurovascular manifestations* , *antiphospholipid syndrome and cerebral venous thrombosis* , *antiphospholipid syndrome and secondary stroke prevention* , and *antiphospholipid syndrome and cognitive impairment* .

This strategy aimed to retrieve literature specifically addressing the domain of APS management and their association with patient outcomes. Key publications outside this timeframe were also considered. Studies with higher levels of evidence, such as randomized controlled trials and meta-analyses, were prioritized for inclusion. Only those published in English were considered. The selection period aimed to highlight the latest and most pertinent advancements in APS. Moreover, we manually reviewed the references of chosen articles to identify additional studies meeting our inclusion criteria.

### Inclusion and exclusion criteria

We included studies that clearly documented clinical outcomes.

Conversely, we excluded studies lacking explicit information on clinical outcomes related to treatment, and those unrelated to neurovascular manifestations associated with APS.

## RESULTS

### Ischemic stroke

Stroke and transient ischemic attack (TIA) are highly prevalent among patients diagnosed with APS 4 ^,^ ranging from 19 to 28% in prospective studies, and are considered the most common arterial complications in APS. 6 7

In a prospective registry of 1000 patients with 10-year follow-up, a combined incidence of stroke and TIA in APS reached approximately 30%. 6 Similar findings were published in a Chinese prospective registry which showed that almost 26% of patients developed stroke or TIA. 8

Identification of antiphospholipid antibodies in blood testing has been associated with a higher risk of stroke as well, even though such patients may have oligosymptomatic or undiagnosed APS. 9 10

More recently, antiphosphatidylserine antibodies have also been found to be associated with worse clinical and functional outcomes with stroke patients, although they are not part of the APS criteria. 11

In terms of etiology, arterial thrombosis, considered the prevailing mechanism, predominantly manifests in the occlusion of large intracranial arteries. Other mechanisms include embolism stemming from secondary valvular heart disease due to APS (Libman–Sacks endocarditis), chronic occlusive vasculopathy affecting small- and medium-sized intracerebral arteries, lesions of the extracranial carotid artery, inflammatory vasculitis of intracranial arteries, cervical artery dissection and Sneddon syndrome. 8 12 13 14 15 16 17 Very few data are available regarding the safety of intravenous thrombolysis in APS patients, although a few case reports show evidence of generally asymptomatic hemorrhagic transformation. 18 19 20

### Stroke prevention

While randomized data on optimal preventive measures for ischemic stroke in this population are limited, some key findings are important to highlight. The Rivaroxaban in APS study (RAPS) was a phase ⅔ randomized controlled trial RCT that failed to demonstrate noninferiority of rivaroxaban 20 mg daily when compared with standard VKA (INR: 2.5) in reducing percentage changes in endogenous thrombin potential (ETP) from randomization to day 42, and a difference in thrombotic risk was not demonstrated between groups. 21 The trial of rivaroxaban in APS (TRAPS) study was another noninferiority multicenter trial in APS patients with a previous thrombotic episode that compared rivaroxaban 20 mg daily to standard VKA to prevent thromboembolic events as measured by a composite outcome of any thromboembolic events, major bleeding, and vascular death. 22 The trial did not reach the noninferiority margin and, in fact, was stopped prematurely due to safety concerns in the rivaroxaban group, with a 19 versus 3% rate of thromboembolic events, major bleeding, and vascular death favoring VKA. This resulted in a number needed to harm of 6.25 (HR = 6.7; 95% CI: 1.5–30.5). 22 Interestingly, 4 (7%) patients in the rivaroxaban group had a stroke compared with none in the warfarin group. It is important to note that, in the TRAPS trial, all individuals included were triple-positive for APA, so results may not be extrapolated in other APA populations.

Similar results were found in another trial, in which rivaroxaban was found not be noninferior when compared with standard VKA. 23 After a 3-year follow-up, recurrent thrombosis occurred in 11 patients (11.6%) in the rivaroxaban group and 6 patients (6.3%) within the VKA group (RR 1.83; 95% CI: 0.71–4.76). Furthermore, stroke was more common among patients administered rivaroxaban (9 events) compared with KAs (0 events), with a corrected relative risk (RR) of 19.00 (95% CI: 1.12–321.9) while major bleeding events were similar among groups. 23

The pilot ASTRO-APS trial aimed to compare apixaban with VKA to prevent thrombosis in thrombotic APS. Although it was terminated early due to inadequate patient accrual, 6 (26%) patients in the apixaban group experienced stroke versus none in the warfarin group. 24 25 As a whole, current randomized data do not support the use of DOACs as a safe secondary stroke prevention option in APS patients, particularly in higher risk triple-positive patients. Therefore warfarin (INR: 2–3) remains the mainstay of treatment to prevent thrombotic events in these patients. 26

While evidence suggest that warfarin offers the best protection against thrombotic events in APS, some studies have examined whether a higher INR (3.1–4.0, target: 3.5) may be more beneficial than standard target (2.0–3.0, target: 2.5). Crowther 27 and Finazzi 28 assessed recurrence of thrombotic events and vascular death or major thrombosis, respectively, but failed to demonstrate the superiority of high-dose VKA when compared with standard dose. Notably, the number of events was low (3 vs. 2) during a mean of 2.7 and 3.4 years of follow-up, respectively. Furthermore, a meta-analysis of these studies' results, with a total of 223 participants, showed a relative risk reduction of 1.37 (95% CI: 0.26–7.12; I2 = 0%; low-certainty evidence). 29 Adding antiplatelets to standard warfarin was also tested as a possible approach, with no significant differences found in stroke recurrence. 29

Hydroxychloroquine (HCQ), a classic antimalarial medication, used for decades for inflammatory diseases such as SLE, seems to have a good safety profile and may also have a role in thrombotic prevention in addition to already known vasoprotective effects with SLE and rheumatoid arteritis. 30 31 A retrospective analysis of nonrandomized data from China demonstrated a significantly likely protective effect from HCQ (OR: 0.549, 95% CI: 0.316–0.952, *p* = 0.033) with regards to stroke. 8 Whether this can translate into clinical benefits is still unknown, given the absence of randomized data, although this area is ripe for further research.

### Moyamoya syndrome

Moyamoya syndrome (MMS) is a progressive cerebral vasculopathy characterized by terminal carotid artery stenosis and pathologic neovascularization in the lenticule-striate territory. It may be associated with both ischemic and hemorrhagic strokes. 32

Antiphospholipid syndrome and MMS are two distinct medical conditions that can occur simultaneously, leading to complex clinical presentations. The majority of the published literature includes case reports; however, it appears to be consistent that both diseases may share physiological features. It is suggested that vascular endothelial damage and secondary thrombosis to altered flow, as well as prothrombotic features related to APS, may lead to Moyamoya syndrome. 33 34

Interestingly, while both diseases may increase ischemic stroke risk, secondary stroke prevention can be quite challenging. On the one hand, MMS is associated with a higher risk of intracranial hemorrhage, on the other hand, standard treatment for APS includes anticoagulation, which increases the risk of ICH. A careful balance of risk and benefits must be made when selecting optimal treatment for patients with both diseases. 33 34 35

### Cerebral venous thrombosis

Cerebral venous thrombosis (CVT) is a rare disease that commonly associated with thrombotic conditions, with APS being responsible for 4 to 10% of all cases. 29 30 31 In the previously described RCTs studying patients with APS, no cases of CVT were described during follow-up. A similarly low risk was found in a cohort of 1,000 patients, with only 7 (0.7%) cases diagnosed over a 10-year follow-up period. 6 21 22 23 29

Although rare, CVT can also be related to APS. 36 Furthermore, in a secondary analysis of the international ACTION-CVT registry, the presence of ≥ 1 positive APA was shown to be a risk factor for recurrence (aHR 3.85; 95% CI: 1.97–7.50; *p* < 0.001). 37

The RESPECT-CVT 38 and SECRET trials 39 as well as the recent ACTION-CVT registry, 40 have opened for the door to the use of DOACs as an initial treatment, which are now suggested by the American Heart Association and American Stroke Association and the Canadian Stroke Best Practices Recommendations 41 as a reasonable option for this condition. 42 Although DOACs show promise for CVT, APS patients were excluded from these trials, so it would be premature to recommend DOACs in these patients.

### Cognitive impairment

Beyond overt neurovascular manifestations, cognitive impairment is also seen in APS. 12 The underlying pathophysiology might be a result of a chronic micro-thrombosis in the smaller arterioles found in deep brain structures, which may induce cerebral microangiopathy and subsequent neuronal damage, leading to a cognitive dysfunction, although inflammation without ischemia may also play a role. Studies have shown that cognitive impairment affects a significant proportion of APS patients, ranging from 15 to 42%. 5 43 The severity and frequency of cognitive impairment correlate with the presence of antiphospholipid antibodies, particularly high titers, and multiple antibody types. 44 Cognitive domains commonly affected in APS patients include executive functioning, memory, visuospatial ability, verbal fluency, and attention. 45 46

While the exact pathophysiology of cognitive impairment in APS is not fully understood, it appears to involve both thrombotic and nonthrombotic mechanisms. Events resulting from antiphospholipid antibodies contribute to microvascular thrombosis and subsequent brain injury with consequent brain atrophy. 43 However, nonthrombotic mechanisms, such as inflammatory processes driven by direct binding of antiphospholipid antibodies to brain tissue, may also play a significant role. 45 White matter disease may be found in APS patients and could be one possible mechanism for cognitive impairment within this group. 47 48

Randomized available data from the previously mentioned RCTs did not capture whether individuals had any cognitive impairment or not.

Future research should focus on using brain imaging biomarkers to better understand and predict cognitive impairment among APS patients and, ultimately, determine whether treatments like anticoagulation and immunosuppression could be helpful in preventing or decreasing it.

### Sneddon syndrome versus APS

Sneddon syndrome is characterized by a combination of cerebrovascular clinical ischemic events and radiological infarcts leading to progressive cognitive impairment, with the presence of diffuse livedo reticularis over the skin. Antiphospholipid antibodies may be present or not in these patients. 49 50 Despite overlapping clinical features, there is distinct clinical and laboratory evidence delineating APS and Sneddon syndrome as different entities, since over half of the latter have been reported with absent antiphospholipid antibodies. 51 Additionally, the clinical courses are different, with Sneddon syndrome having a more progressive clinical course and a poorer neuropsychiatric prognosis. 51 52

In conclusion, as such, APS presents a complex interplay of neurovascular manifestations, such as ischemic stroke, cerebral venous thrombosis, and cognitive impairment. While the pathophysiology of related neurovascular complications is multifactorial, the presence of antiphospholipid antibodies highly correlates with the development of these conditions. Current evidence underscores the high prevalence of stroke among these patients, emphasizing the importance of effective secondary prevention strategies.

Trials evaluating the use of DOACs in APS-associated stroke or CVT are lacking and those that have been published have raised safety concerns for the use of such drugs among APS patients. Therefore, caution is advised until further research is conducted. As a result, VKAs remain the mainstay treatment for related strokes. Additionally, cognitive impairment represents a considerable burden in APS patients, necessitating further investigation into optimal management approaches. Overall, a comprehensive understanding of this condition and its neurovascular implications is crucial for guiding clinical decision-making and improving patient outcomes.
